# Characterisation and correction of signal fluctuations in successive acquisitions of microarray images

**DOI:** 10.1186/1471-2105-10-98

**Published:** 2009-03-30

**Authors:** Annie Glatigny, Hervé Delacroix, Thomas Tang, Nicolas François, Lawrence Aggerbeck, Marie-Hélène Mucchielli-Giorgi

**Affiliations:** 1 Centre de Génétique Moléculaire, CNRS FRE3144, F-91198 Gif-sur-Yvette, France; 2 Université Paris-Sud 11, F-91405 Orsay, France; 3 Université Pierre et Marie Curie – Paris 6, F-75005 Paris, France; 4 TELECOM & Management SudParis, 9 rue Charles Fourier, F-91011 Evry, France

## Abstract

**Background:**

There are many sources of variation in dual labelled microarray experiments, including data acquisition and image processing. The final interpretation of experiments strongly relies on the accuracy of the measurement of the signal intensity. For low intensity spots in particular, accurately estimating gene expression variations remains a challenge as signal measurement is, in this case, highly subject to fluctuations.

**Results:**

To evaluate the fluctuations in the fluorescence intensities of spots, we used series of successive scans, at the same settings, of whole genome arrays. We measured the decrease in fluorescence and we evaluated the influence of different parameters (PMT gain, resolution and chemistry of the slide) on the signal variability, at the level of the array as a whole and by intensity interval. Moreover, we assessed the effect of averaging scans on the fluctuations. We found that the extent of photo-bleaching was low and we established that 1) the fluorescence fluctuation is linked to the resolution e.g. it depends on the number of pixels in the spot 2) the fluorescence fluctuation increases as the scanner voltage increases and, moreover, is higher for the red as opposed to the green fluorescence which can introduce bias in the analysis 3) the signal variability is linked to the intensity level, it is higher for low intensities 4) the heterogeneity of the spots and the variability of the signal and the intensity ratios decrease when two or three scans are averaged.

**Conclusion:**

Protocols consisting of two scans, one at low and one at high PMT gains, or multiple scans (ten scans) can introduce bias or be difficult to implement. We found that averaging two, or at most three, acquisitions of microarrays scanned at moderate photomultiplier settings (PMT gain) is sufficient to significantly improve the accuracy (quality) of the data and particularly those for spots having low intensities and we propose this as a general approach. For averaging and precise image alignment at sub-pixel levels we have made a program freely available on our web-site  to facilitate implementation of this approach.

## Background

Two color microarrays allow massively parallel profiling of gene expression in a single hybridization experiment [1,2]. The study of two different mRNA populations consists of 1) labelling each set of transcripts with a fluorescent dye, usually Cy3 and Cy5, 2) challenging the transcripts in a competitive hybridization towards thousands of specific gene probes spotted on an array and 3) measuring the fluorescence signals for each dye over the whole array. The two 16 bit images produced by the scanner are combined and the data for each spot are extracted and processed to yield ratios between the tested conditions. These ratios are used for further analysis. This technique, however, suffers from an excess of variability that limits the measurements' robustness. Uncertainties in the measurements come from different sources among which are data acquisition and image processing [3,4].

In the microarray approach, the final interpretation of the experiments strongly relies on the accuracy of the measurement of the signal intensity at each spot. Therefore, array scanning and image processing are crucial steps that need to be optimized thoroughly. Scan parameters depend upon the instrument and upon user-controlled settings (for review see Timlin [5]). Since a single scan is usually not sufficient to provide all the statistically significant information that is available on the slide, different protocols employing multiple scans have been proposed. Dudley et al [6] combined multiple scans performed at different and linearly spaced sensitivity settings. Other authors have proposed the computation of data from scans acquired at multiple scanner sensitivity settings (two or more PMT gains) [7-9]. Variability among microarray images also has been studied by Tang et al. [10] and Romualdi et al. [11]. Their results showed that, following independent scans, a single pixel belonging to a given spot can have different levels of fluorescence intensity. They, also, have shown that DNA spot images scanned with the same settings (resolution and PMT) are not exactly superimposed. They have proposed solutions either to correct image misalignment or to reconstitute one single virtual image statistically that is representative of a series of consecutive scans of a microarray (Σ POT software). Romualdi et al. demonstrated that the use of multiple scans 1) reveals false positive results such as differentially expressed genes that are detected by a single scan but not confirmed by successive scanning replicates 2) increases the image homogeneity and 3) enhances the detection of differentially expressed genes, particularly for genes with a low level of expression. Indeed, when the fluorescence level of a given spot is close to the background, it is usually rejected from further statistical analysis [12,13]. However, these authors did not analyse, among successive scans, the fluctuations of individual pixel intensities or median spot intensities. Since the intensities of the weak spots are often too variable among replicated slides to allow the signal to be considered as statistically relevant, the associated genes are not selected as being significantly differentially expressed.

A previous study by Romualdi et al. [11] was limited to series of consecutive scans obtained with the same scanning parameters. Here, we have investigated the effect of PMT settings and scanning resolution on the variability of the fluorescence signal among successive scans for the same array. To avoid any specific influence resulting from comparison of different experimental conditions, we used data from arrays for which self-to-self hybridizations of two identical samples from various species was performed. Since the photo-bleaching of each dye is low, this allowed us to study the fluctuation of the spot median at different intensity levels. For a given spot, we determined the standard deviations of the pixels along the series of scans, to assess which part of the spot is responsible for the fluctuations of the intensity median from one scan to the next. Finally, for one array that contained repeated probes (the same probe is present two times on the array to increase the quality of the data; as shown in references [14] and [15]) and which had been hybridized with two biological samples, we determined the effect of averaging successive scans on the reproducibility of the signal of the duplicated genes.

Our results show that averaging two image acquisitions of the same array is sufficient to enhance the robustness of the value of the signal, particularly in the case of weak spots, and we propose a new procedure to enhance the detection of differentially expressed genes.

## Results

### Analysis of the decrease in the signal

One problem that can arise when microarrays are submitted to repetitive scans is photo-bleaching. To evaluate this phenomenon, we compared the median of the intensities from the first scan in a series to the last scan, the series being scanned with an Axon scanner at PMT settings of 400 V for Cy5 and 400 V for Cy3. The result of this comparison reveals that the decrease in the signal is small (0.5% from one scan to the next for the red dye (Cy5) and 0.1% for the green dye (Cy3)) Our results are in agreement with those of Bengtsson et al [16]. However, we noticed that this decrease is not regular from scan to scan along each series, particularly in the green channel. To render the images within a series comparable, this irregular decrease has to be corrected. To do so, we adjusted the F635 and F532 median values as described in the materials and methods.

### Influence of the scanning parameters on the signal variability

Once the effect of the decrease in the signal was corrected, we were able to study the influence of other parameters such as the scanning resolution and the PMT settings on the signal variability.

#### Effect of the scanning resolution

Technical progress in array manufacturing has permitted an increase in the density of the spots present on a slide. However, the size of the spots, and, thus, the number of pixels per spot, has decreased. Further, the number of pixels is directly related to the scanning resolution. For example, a spot with a diameter of 100 microns is composed of 80 pixels at a scanner resolution 10 microns and four times more pixels when the resolution is 5 microns, (approximately 320 pixels). It is not known to what respect the signal associated with a spot is affected by the resolution of the scan. To investigate this relationship, we compared the signals obtained at 5 and 10 microns scanner resolution from two high density arrays having spots with mean diameters of 100 microns (slides #1 and #2, Table 1). The criterion chosen to measure the signal variability is an adjusted signal that varies more than 10% between two consecutive acquisitions (see materials and methods). The percentage of "outlier" spots corresponds to the percentage of spots whose adjusted signal satisfies this criterion. We chose this threshold since it permits the detection of small fluctuations while still maintaining enough spots for a robust statistical study. For example, at PMT settings of 700 V in both channels and 10 microns resolution, 4% of the spots have fluctuations higher than 10% but only 1% present fluctuations higher than 12% whereas for 86% of the spots the fluctuation is higher than 7%. We found that the percentage of "outlier" spots is higher at 10 microns resolution than at 5 microns. Indeed, because of the reduced number of pixels per spot at 10 microns, the robustness of the intensity value (median value) is reduced. This is true for both the red and the green channels, but the percentage of "outlier" spots is higher for Cy5 than for Cy3 (see Table 2).

**Table 1:** List of the microarray types

Slide #	Reference	Manufacturer	Genome	Number of spots	Surface chemistry	Labelling system	Scan resolution	Auto-PMT
1	Custom	Agilent technologies	fungus	44290	phosphoramidite	(Cy-aRNA) LRILAK	5/10 microns/pixel	n.d.

2	Custom	Agilent technologies	fungus	44290	phosphoramidite	(Cy-aRNA) LRILAK	5/10 microns/pixel	n.d.

3	Catalog (# maize-45 k1)	University of Arizona	plant	46128	aminosilane	(Cy-aRNA) LRILAK	10 microns/pixel	700 V Cy5 600 V Cy3

4	Custom	CEA, Evry	archea	4608	hydrogel matrix	Cy-cDNA (SuperScript Indirect)	10 microns/pixel	600 V Cy5 500 V Cy3

5	Catalog (#G4140B)	Agilent technologies	yeast	10807	phosphoramidite	(Cy-aRNA) LRILAK	5 microns/pixel	650 V Cy5 500 V Cy3

Descriptive list of microarray types and corresponding procedures used in the experiments included in this study, n.d. not done.

**Table 2:** Percentage of "outlier" spots as a function of the PMT gain and the resolution.

	**5 microns resolution**		**10 microns resolution**	
PMT voltages	F635	F532	F635	F532

400-400	0.0	0.0	0.0	0.0

500-500	0.0	0.0	0.7	1.4

600-600	1.4	0.5	6.5	3.2

700-700	3.7	1.8	11.5	4.0

Results for slide #1. 400-400 corresponds to settings 400 V for the red dye (F635) and 400 V for the green dye (F532), respectively. The percentage of "outlier" spots, *P*_*out*_, is equal to: *P*_*out *_= *N*_*out*_/*N *with *N*_*out *_being the number of events where a spot is found to be an "outlier" between two successive scans and *N *= (*Nscans *-1) × *n*_*spots *_(*Nscans *is the number of scans in the series and *n*_*spots *_is the number of spots on the slide).

#### Effect of the PMT settings

**Figure 1:**
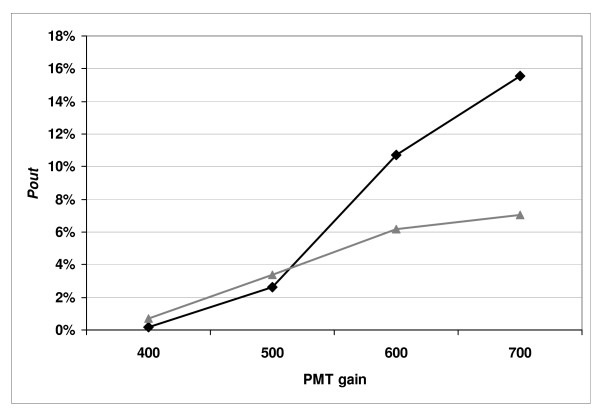
**The percentage of "outlier" spots, *P*_*out*_, is plotted versus PMT gain in slide #3**. *P*_*out *_= *N*_*out*_/*N *with *N*_*out *_being the number of events where a spot is found to be an "outlier" between two successive scans and *N *= (*Nscans *-1) × *n*_*spots *_(*Nscans *is the number of scans in the series and *n*_*spots *_is the number of spots on the slide). The lines with triangles are those obtained for the Cy3 signal (532 nm) and the lines with lozenges are for the Cy5 signal (635 nm).

We also found that the fluctuation of the signal increases as the PMT voltage increases (see Table 2 and Figure 1) except for the hydrogel slide where the percentage of "outlier" spots was constant in the green channel at all the PMT settings. When the PMT gain increased from 400 V to 700 V, the signal fluctuations for each PMT setting were different depending upon the surface chemistry. For the hydrogel slide (#4), the percentage of "outlier" spots was very low (from 0 to 0.2% and from 0 to 4% in the green and the red channels, respectively). For the aminosilane slide (#3) it increased from 0 to 7% and from 0 to 15% in the green and the red channels, respectively. For phosphoramidite slides (#1, #2) the percentage of "outlier" spots, at 10 microns resolution, increased from 0 to 4% and 0 to 12% in the green and the red channels, respectively (Table 2).

**Figure 2:**
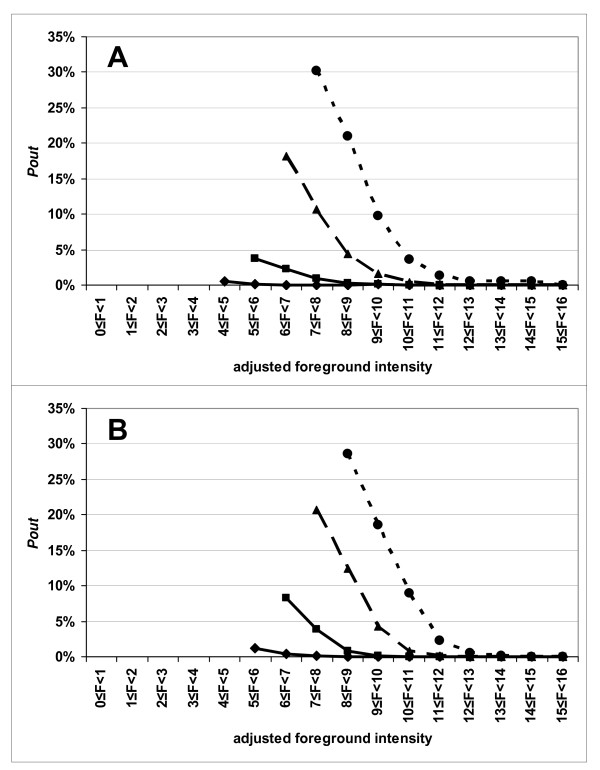
**The percentage of "outlier" spots, *P*_*out*_, is plotted versus the intensity class and PMT gain**. Results for slide #3. The percentage of "outlier" spots in each intensity class  is *P*^*c*^_*out *_= *N*^*c*^_*out*_/*N*^*c*^, with *N*^*c*^_*out *_being the number of events where a spot in the intensity class, *c*, is found to be an "outlier" between two successive scans and *N*^*c *^= (*Nscans *-1) × *n*^*c*^_*spots *_(*Nscans *is the number of scans in the series and *n*^*c*^_*spots *_is the number of spots in the intensity class *c*). The line with the lozenges is obtained for PMT gains 400-400 (Cy5-Cy3); the line with the squares is for PMT gains 500-500; the line with the triangles is for PMT gains 600-600 and the line with the dots is for PMT gains 700-700. Part A is for the Cy3 image (532 nm); Part B for the Cy5 image (635 nm).

In order to study the relationships among these fluctuations and the signal levels, for each PMT gain, we computed the number of "outlier" spots according to their intensity class (there are 16 classes of intensities, corresponding to the log_2 _of the green or the red intensities). For each channel, the lower the intensity of the signal, the higher the number of "outlier" spots (Figure 2). As described above, for each intensity class, the percentage of "outlier" spots increases with the PMT settings and it is higher for the red dye than for the green dye. For example, for slide #3, the percentage of "outliers" is 1.6% (160 spots) at PMT settings of 600 V and 9.7% (2467 spots) at PMT settings of 700 V for the spots having a log_2 _of the green fluorescence level between 9 and 10. The percentage of "outliers" is 0.4% (22 spots) at PMT settings of 600 V and 3.6% (500 spots) at PMT settings of 700 V for the spots having a log_2 _of the green fluorescence level between 10 and 11. There were twice as many "outlier" spots for the red dye as for the green dye.

These signal fluctuations associated with each spot in the red or the green channel, especially with increasing PMT gain (greater than 600 V), impact on the red/green ratios and, thus, influence the differential analysis. These observations indicate that scanning should be performed with moderate PMT settings. However, this is in opposition to the necessity of finding spots in all intensity classes for which increasing the settings increases the range of fluorescence values for the spots and, thus, improves spot detection. Different authors have proposed combining scans at low and high sensitivities to increase the number of spots detected on an array [5-8]. We evaluated an alternative protocol consisting of performing consecutive scans at the same PMT settings and then calculating the mean of 2 or 3 successive scans.

### Effect of averaging successive acquisitions

#### Effect on the signal variability

Some scanners, such as the Axon scanner (one of the most frequently used in the microarray field), offer the possibility of acquiring each line of the array several times (*LS *mode, see material and methods) and computing the arithmetic mean of these acquisitions. We performed a series of experiments using this option. Slides were scanned at balanced voltages in order to determine the best parameters for acquisition of the images. The images were aligned at sub-pixel levels and analysed as previously described [10].

**Figure 3:**
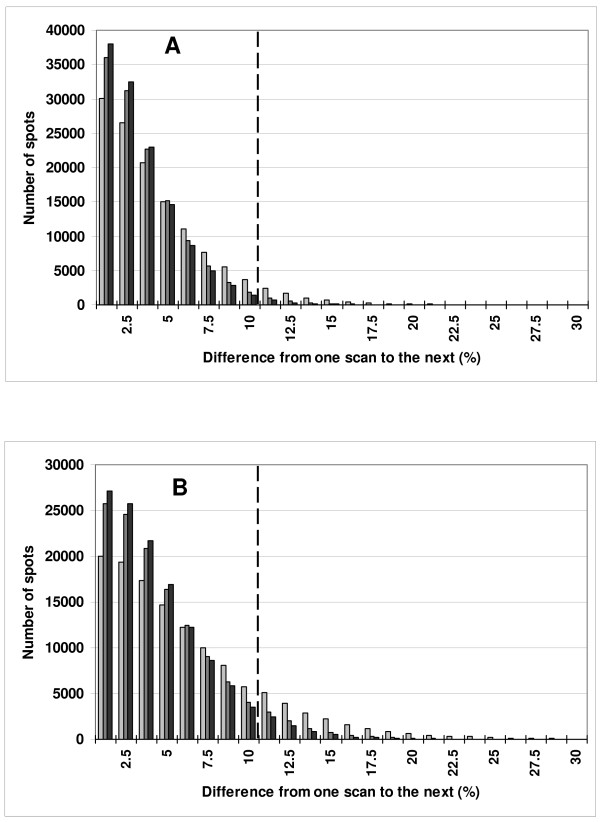
**Distribution of spots as a function of the relative difference between two scans**. The number of spots having a difference of intensity from one scan to the next in a series of eight successive scans (see *P*_*f, s, n *_in materials and methods) is plotted as a function of that difference, for slide #*3*. Each series of bars in the histogram increases by a relative difference of 1.25 percent of the adjusted intensity. Bars in light grey represent the results for images with one acquisition; bars in grey represent those for images obtained with two acquisitions of the same line and those in black for images obtained with three acquisitions. The broken line corresponds to a difference of ten percent from scan to scan. Part A is for Cy3 images (532 nm); part B for Cy5 images (635 nm).

**Figure 4:**
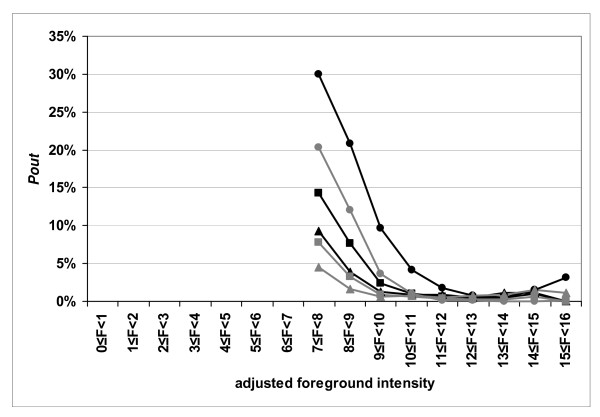
**The percentage of "outlier" spots, *P*_*out*_, is plotted as a function of the adjusted foreground intensity**. Results for slide #3. The percentage of "outlier" spots in each intensity class, , is calculated as follows *P*^*c*^_*out *_= *N*^*c*^_*out*_/*N*^*c*^, with *N*^*c*^_*out *_being the number of events where a spot, in the intensity class, *c*, is found to be an "outlier" between two successive scans and *N*^*c *^= (*Nscans *-1) × *n*^*c*^_*spots *_(*Nscans *is the number of scans in the series and *n*^*c*^_*spots *_is the number of spots in the intensity class). The value of the signal that is used to select the "outlier" spots is the average of the adjusted foreground signals obtained for 1, 2 or 3 acquisitions per line. The grey lines correspond to the Cy3 image (532 nm) and the black lines to the Cy5 image (635 nm). The lines with the circles correspond to one acquisition per line; those with squares to two lines averaged and those with triangles to three lines averaged.

The number of "outlier" spots, was found to decrease with an increase in the number of scans per line. This was more pronounced for the red channel, where the percent of outlier spots decreased from 16.7% to 5.0%, than for the green channel where the decrease was from 6.4% to 1.5% (Table 3). In Figure 3, for the green channel (panel A) and for the red channel (panel B) the number of spots presenting large differences decreases when the number of acquisitions increases. Whatever the intensity class or the dye, the number of "outlier" spots decreased when 2 or 3 acquisitions were used to compute the average for a line, except for the log_2 _of intensities between 14 and 16, where there were very low numbers of spots (Figure 4). This decrease was more pronounced going from 1 to 2 acquisitions as opposed to that going from 2 to 3 acquisitions. Moreover, the decrease in the percentage of "outliers" is more pronounced for spots with low intensity values, decreasing for example, from 30.0% to 14.3% and then to 9.3%, respectively when 2 or 3 acquisitions are averaged, for red intensities having a log_2 _value between 7 and 8. Thus, averaging leads to increased levels of confidence in the values of the signals, whatever the signal level. This results in the inclusion of more low intensity spots in the differential analysis.

**Table 3:** Percentage of "outlier" spots as a function of the number of acquisitions for a line.

Averaged lines	F635	F532
1	16.7	6.4

2	7.0	2.0

3	5.0	1.5

Results for slide #3. The PMT settings were 700 V for Cy5 (F635) and 600 V for Cy3 (F532); images were acquired at 10 microns resolution using the LSmode (see materials and methods).

#### Effect on the variability of the intensity ratios within the series of scans

Although these results illustrate how the quality of the signal can be increased by averaging consecutive scans, they do not address the consequences on the log_2 _ratio values. To elucidate this aspect, we performed an analysis of the fluctuations of the M values (M is the *log*_2 _ratio of green and red intensities) within a series of *Nscans*. We computed, for each interval of the A value (A is the geometric mean of the *log*_2 _green and red intensities), a parameter *e*. This parameter is a measure of the fluctuation of the log_2 _red/green ratios within the series, for all "outlier" spots (see Additional file 1) on the one hand and for the "non-outlier" spots on the other hand (results not shown). The results were very different between "outlier" and "non-outlier" spots, as the "non-outlier" spots showed constant fluctuations within a series independent of the A values. The difference between the fluctuations of the M values of "outlier" and "non-outlier" spots decreased with the number of acquisitions used for averaging. This decrease was larger when going from 1 to 2 scans than for 2 to 3 scans. Moreover, the strongest effect was seen for the spots in the low intensity classes. Thus, averaging consecutive scans results in a decrease in the fluctuation of M within the series, especially for the low intensity spots.

#### Effect on the pixel variability

**Figure 5:**
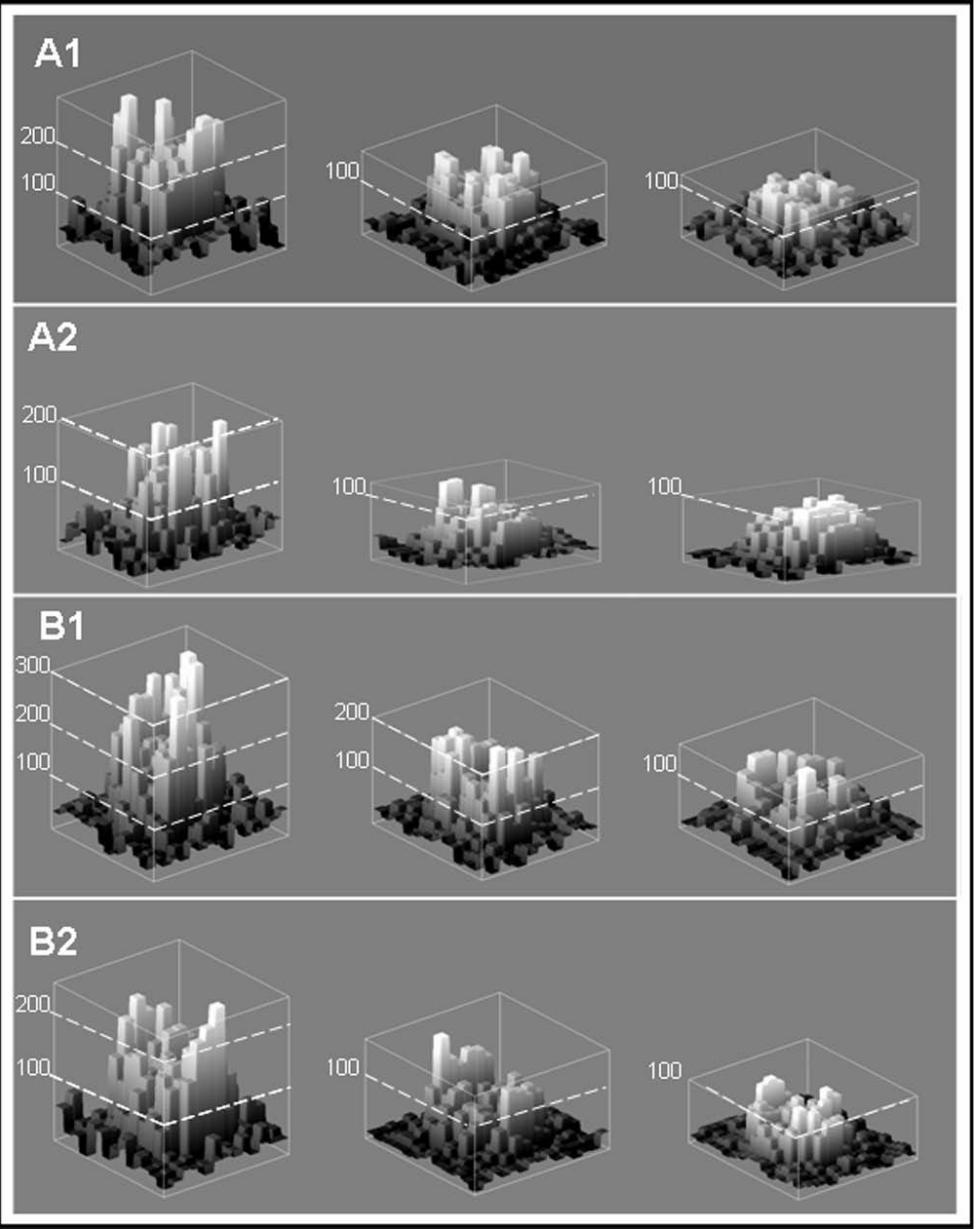
**The standard deviation of each pixel of "non-outlier" spots and of "outlier" spots**. Spots derived from 8 successive scans with one, two or three acquisitions per line are shown from the left to the right. The height of each vertical bar corresponds to the standard deviation of one pixel within the scan series, graduations are indicated by white broken lines. Parts A1 and A2 are for "non outlier" spots of intensity *F635*_*adj *_equal to 1100 and *F532*_*adj *_equal to 1050, respectively; B1 and B2 are for "outlier" spots of intensities 1100 and 1050, respectively. For "outlier" spot B1 the maximum of the standard deviation decreases from 300 to 200 to 160 while in A1 it is lower and decreases from 280 to 160 to 100 going from one to three successive acquisitions. For "outlier" spot B2, the maximum of the standard deviation decreases from 240 to 200 to 160 while in A2 it is lower and decreases from 200 to 120 to 100 going from one to three successive acquisitions.

To understand the reason for the fluctuations observed within a series, we analysed spots at the pixel level. Spots can have different shapes (donuts, craters, etc); they can have irregular contours. When the adaptative circle method of segmentation is used, all the pixels of the contour do not have the same intensities. To visualize the behaviour of the pixels, the *Nscans *aligned images of the same series were stacked. One "non-outlier" spot and one "outlier" spot having the same intensity levels (*F532*_*adj *_equal to 1050 for the spots A2 and B2 on Figure 5, and, *F635*_*adj *_equal to 1100 for the spots A1 and B1) were selected and the images were cropped. We calculated the mean and the standard deviation of the intensity of each pixel in these stacks. The resulting images (Figure 5 and Additional file 2) were processed with an Image J plug-in adapted from "interactive 3D surface plot" [17]. Comparison of "outlier" and "non-outlier" spots shows that the standard deviation of "outlier" spots is always higher. For the example of spots B1 and A1, the maximum values of the standard deviation are equal to 300 and 280, respectively. These values are remarkably high compared to the intensity levels of the spots (*F635*_*adj *_equal to 1100). In addition, these large fluctuations concerned many pixels; for the "outlier" spot B1, 95% of the pixels have a standard deviation greater than 100. Since our definition of an "outlier" spot is based upon a fluctuation of the median intensity greater than 10% from one scan to the next, this indicates why the pixels of "outlier" spots are more variable from scan to scan. Indeed, as the median is a measure insensitive to the extreme values, the median of pixel values of each spot changes from one scan to the next, only when a large number of pixels vary to a large extent with respect to the previous scan.

The data shown in Figure 5 are in agreement with the expected results and demonstrate the heterogeneity of the spots. We observed that the pixels of the contour of the spots vary more than the pixels in the middle of the spots. Averaging scans decreased the value of the standard deviation of all the pixels within the series (for example in Figure 5, the maximum is 160 for "outlier" (B2) and 120 for "non-outlier" spots (A2) with 2 acquisitions averaged; it is 100 with 3 acquisitions averaged for both spots).

#### Effect on the reproducibility of the signal and of the M values

**Figure 6:**
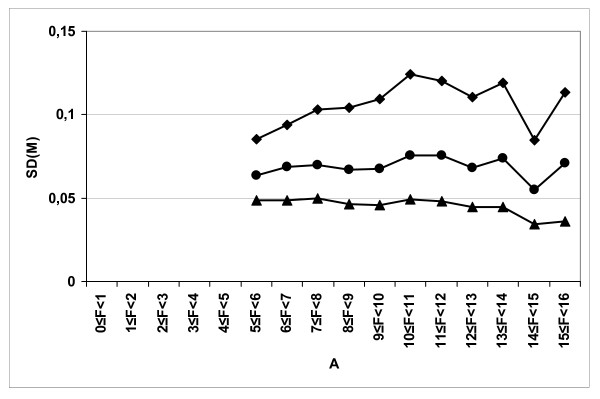
**The standard deviation of the M values, SD(M), of replicated spots in slide *#5***. The standard deviation of the M values is computed as a function of the A values. The line with the lozenges corresponds to the images obtained with one acquisition per line; the line with the dots corresponds to the images obtained with two acquisitions per line and the line with the triangles corresponds to the images obtained with three acquisitions per line.

Finally, we wanted to determine the impact of averaging consecutive scans on the robustness of the measurement of the signal and on the difference in expression, as evaluated by the reproducibility of both measures. To do that, we analyzed slide *#5 *(see Table 1) which was a competitive hybridization between two different biological samples. There are several levels of replication (biological, technical, spot replication, multiple scans) which improve the quality of the measurement and which allow an evaluation of the variance of the data (for review see Karakack [18]). Two thirds of the spots on the array of slide *#5 *are randomly replicated. The array was scanned using the *LS *mode (see material and methods). We observed a large decrease in the standard deviation of the log-ratios (M values) (see Figure 6) of replicated spots when the number of acquisitions used for averaging increased, whatever the class of intensity. Within a series, the deviation between the log-ratio values attained 0.1 or 0.12. The majority of the log-ratio values (more than 99%) vary from -1 to +1, so these observed deviations are high. We observed that 8% of the duplicated spots were significantly different (p < 0.05) but only 1/4 of them are "outlier" spots, either in F532 or F635. This percentage decreases to only 1% after averaging 2 acquisitions and remains at 1% after averaging 3 acquisitions. As noted previously, in the majority of the analyses, weak spots are often filtered. This leads to the elimination from the analysis of potentially interesting genes [13]. Decreasing of the log_2_-ratio variability of the intra-array replicates has, as a direct consequence, the reduction of the inter-array log_2_-ratio variability. We have shown that this reduction of variability exists for spots with weak intensities and, thus, the genes having low levels of expression in one of the conditions to be compared can be detected better.

## Discussion

The observed signal intensity of the array results from several parameters extending from the hybridization of the target and the probe to the scanning. Using an Axon 4000B scanner, we have demonstrated that the behaviours of the spots on an array are not identical. Some spots display more fluctuations than others and, moreover, the green and the red dyes do not have the same behaviour.

Our results show that, for a series of successive scans, the decrease of the signal is weak but irregular. The variation in intensities involves many spots and is higher for the red channel as opposed to the green channel. The variation increases with increasing PMT gain and is higher for the lower intensity signals. Thus, when the gain is increased, the detection of spots is improved but the fluctuations of the signals increases. These observations suggest that scanning should be performed with moderate PMT settings. However, this is in opposition to the need for finding and measuring accurately the intensities of spots in all of the intensity classes and, particularly, in the low intensity classes. Considering this paradox, a good compromise would be to scan arrays with the highest PMT setting for which there are no "saturated" spots. However, to increase spot detection, different authors have proposed combining scans at low and high PMT settings [6-9] and/or using a method to calibrate dye biases [16]. At high PMT settings, red signal values are more variable so their log_2_-ratios are less accurate. We propose an alternative protocol consisting of averaging 2 (or 3, as a maximum) consecutive scans. We show that the fluctuation of the signal decreases when the number of scans averaged increases from 1 to 3 (the percentage of "outliers" decreases from 16.7% to 7.0% to 5.0% for the red channel on slide #3, for example). Moreover, the effect of the averaging is most marked for the spots having low intensities.

We found that the fluctuations of the signal are due mainly to a strong texture in the spot (spots are not homogeneous) or, sometimes, to its border, which presents variations greater than the other pixels in the spot. This is especially true when the spots have irregular contours and are segmented with the adaptive circle method. The border of the circle delimiting the spot contains pixels both of the background and of the signal. It might be assumed that, if the circle is completely inside the spot, the number of "outlier" spots would be reduced. But this is not what is observed. Indeed, by reducing the circle size, the number of pixels per spot decreases and the median, computed from all the pixels of the spot, is then less robust. Since the "outlier" spots, are less homogeneous than the other spots, the averages of several consecutive acquisitions will decrease this texture. The number of pixels depends directly on the scanning resolution and, as a consequence, the texture, too. Thus, the signal fluctuation is higher at 10 microns resolution than at 5 microns. Consequently, the average of 2 or 3 consecutive scans has a greater effect on arrays scanned at 10 microns resolution.

The aim of the method published by Romualdi et al [11] is to reduce the high variability among pixels in the same spot. They stacked 10 consecutive acquisitions of the same array to increase the homogeneity of the pixels in the spots. Thus, this method allows enhancement of the detection of differentially expressed genes. However, it cannot be used practically because it is difficult to implement. Our method, which consists of averaging two or three acquisitions, is based on the same principle, but it is easier to perform, particularly on the Axon scanner where the *LS *mode can be executed automatically. Even when a scanner does not offer this functionality, averaging two or three independent, consecutive acquisitions of the same array leads to a similar result. But, in this case, it is essential that, before averaging, the consecutive acquisitions are aligned at a sub-pixel level [10]. As shown previously, the photo-bleaching is very low and homogeneous for all spots, therefore, it is not necessary to adjust the medians between consecutive scans before averaging them.

Finally, we show that averaging scans leads to a decrease in the variability of the signal of replicate spots and, in particular, that of low intensity spots. The confidence in the values of the weak signals is thus improved, increasing the threshold of the signal/noise ratio and, consequently, allowing the retention of many more weak spots for analysis.

We also found that averaging leads to a decrease in the standard deviation of the M value of replicate spots. Biological and/or technical repeats with less variation will have improved detection of differentially expressed genes. Since weak spots are affected by the reduction of the standard deviation of the M value of the replicates, this will lead to an improvement in the detection of genes having very low levels of expression in one of the experimental conditions being compared.

## Conclusion

By using series of scans of arrays at different PMT settings and by precisely aligning the images, we have evaluated the signal fluctuations from one scan to the next. We established that the variation in the signals is related to several parameters: the resolution, the values of the settings, the dye, the chemistry of the slide and the intensity of the spot itself. We also demonstrated that these fluctuations can be reduced by averaging scans; in this case, the heterogeneities of the spots and the standard deviation of the replicates are reduced. Moreover, the diminution of signal fluctuation leads to more accurate results, especially in the case of weak spots.

Finally, we recommend scanning at moderate PMT gains and averaging two (or, at most, three) scans to increase the reliability of the fluorescence signal. In some scanners this can be done easily by using the "*LS *mode" or it can be done afterward. For this purpose, a program for image alignment at sub-pixel levels and for averaging is freely available on our web-site .

## Methods

### Microarray experiments

To measure the fluctuations in the signal intensities without taking gene expression into account, the images that were analysed were derived from several types of microarrays consisting of "self-to-self" hybridizations of different independent biological samples (for details see below and Table 1). To compare, within the array, the reproducibility of the ratios of the intensities between two biological conditions from one scan to the next, we carried out an experiment in which two different samples were hybridized (slide *#5*).

All the microarrays used in this study were pangenomic long-oligonucleotide (50–70 mers) arrays but they had different surface chemistries. Phosphoramidite arrays, commercial or custom, were manufactured by Agilent Technologies (Santa Clara, CA, USA). Hydrogel slides were distributed by an academic platform (French national microarray production site – CEA – Evry, France). Aminosilane microarrays were produced by an academic platform (University of Arizona, USA).

The biological samples were derived from various organisms (plant, fungus, yeast and Archea) (Table 1). Target preparation, hybridization and washing were done according to the manufacturers' instructions using the Gif/Orsay DNA Microarray platform's ISO 9001 protocols. The labelling was done via a linear amplification of antisense RNA with direct integration of CyDyes using the Low RNA Input Linear Amplification kit (LRILAK; Agilent Technologies). For some samples (Archea), single strand cDNA were synthesized, and then coupled with dyes using a Superscript™ Indirect cDNA labelling system (Invitrogen, Carlsbad, CA, USA). In each case, the labelling efficiency and product integrity were checked according to criteria defined by Graudens *et al. *[19]. Identical amounts of Cy3- and Cy5-labelled targets were mixed according to the manufacturer/distributor's instructions and incubated on the microarray slides for 17 hours at 60–65°C, in a rotating oven, using an Agilent hybridization system (Agilent technologies). The slides were washed and, then, any traces of water were removed by centrifugation at 800 rpm for 1 min or air dried with ozone-free dry air ("canned air").

### Scanning and acquisition of images

The slides were scanned with an Axon GenePix 4000B scanner (Molecular Devices, Sunnyvale, CA, USA) equipped with 532 and 635 nm excitation lasers for Cy3 and Cy5, respectively. This device allows acquisition of both images simultaneously and different modes of scanning: fixed or automatically adjusted settings, averaged line scanning (*LS *mode).

Each slide was scanned at 100% laser power at 5 and/or 10 microns resolution. All the scans were performed the same day, the number of successive scans (*Nscans*) in a series is, thus, limited to 8 per PMT setting. The procedure was as follows: *Nscans *at PMT voltage 400 V for Cy5 and 400 V for Cy3; *Nscans *at 500 V and 500 V; *Nscans *at 600 V and 600 V and finally *Nscans *at 700 V and 700 V. These settings are within the linearity range of the scanner.

For some slides (see Table 1), "AutoPMT" scanning was done, i.e. voltages were automatically adjusted to balance the distributions of the red and the green intensities and to optimize the dynamics of image quantification. The number of saturated pixels was low (less than 0.05%). These images were acquired using the "line scanning" mode (*LS *mode), e.g. each line of the array is scanned 1, 2 or 3 successive times before scanning the next line; from these multiple measurements an arithmetic mean is computed. This "AutoPMT" series was conducted as following: *Nscans *with each line scanned once; *Nscans *with each line scanned twice and averaged and *Nscans *with each line scanned three times and averaged.

### Image registering and spot finding

From one scan to the next, the "red" or "green" images may be slightly shifted and, moreover, for each scan, the two images are not perfectly aligned. We, therefore, corrected the shift between the images by using the program for registering two-color microarray experiments described in the paper of Tang et al [10], allowing the sub-pixel adjustment of all the images of the series at the same time. The green image of the first scan was used as the reference for the alignment of all the green and the red images. The precision was 1/4 pixel for images obtained at 10 microns resolution and 1/8 pixel for those at 5 microns.

The images were analysed with the GenePix 6 software, the segmentation method being the adaptive circle method. In our experiments, the background was very low and was, thus, not subtracted. Not found, saturated and bad (dust, scratch, etc) spots were discarded.

### Adjustment of fluorescence intensity

All the following analyses were carried out with the R statistical package available at .

The median value of pixel intensities was used to quantify the spot intensity. It does not take the extreme values of pixels into account and, thus, it is the most common type of measurement. Within a series of successive scans having the same scanning parameters, spot intensities fluctuate. To compare the values of the median intensities obtained for each spot we adjusted them as follows: , where *F*_*s, n *_is the median intensity (F635 or F532) of the spot, *s*, at scan number, *n*, . *n*_*spots *_is the number of spots and *med*() is the median  value (*n *= 1: *Nscans*). This adjustment allows the comparison of images within a series of successive scans by homogenizing the mean signals  of all scans. The median of the mean signals of the *n *scans (*med*()) is used so that excessively divergent scans are not taken into account.

### Quantification of the signal variability from one scan to the next

To evaluate the variability of the signal measurements between two successive scans we computed for each channel, (*f*), and for each spot, (*s*), a percentage of variation from one scan, (*n*), to the next, (*n+1*): 

When this percentage is larger than 10%, the spot was considered as an "outlier". We chose this threshold since it is a good compromise; it permits the detection of small fluctuations while still maintaining enough spots for a robust statistical study. Therefore, since we performed *Nscans *successive scans, each spot can be an "outlier" between 0 and (*Nscans *-1) times.

In order to study the variations of the signal from one scan to the next as a function of the spot intensity, the number of "outlier" spots was computed, separately, for successive intensity intervals. In theory, the log_2 _of the intensity varies from 0 to 16 but in practice it is never lower than 4.

### Quantification of the instability of the intensity ratios within a series of scans

In microarray experiments the expression difference is usually quantified by the log_2 _intensity ratios (*M *values). To evaluate the instability of the *M *values within a series of scans, we computed *e *for "outlier" and "non-outlier" spots:



*e *is the average, for all the spots, of the larger of the absolute differences between the M values and their means within a series of scans.

The formula is valid for "outlier" and "non-outlier" spots, *n*_*spots *_being, on the one hand, the number of spots considered as being "outliers" and, on the other hand, the number of spots considered as being "non-outliers".

### Analysis of intra-array probe repetitions

Some arrays used in this analysis contained replicated spots. These allow multiple measurements for the same probe in the same hybridization experiment. In yeast microarrarrays from Agilent technologies (slide *#5*), 2/3 of the probes are replicated and spotted randomly (see Table 1). For data coming from scans carried out using the "*LS *mode", the standard deviations of the ,  and of the *M*_*s, n *_values of the replicates for each probe were computed as a whole and, also, computed separately for each class of intensity and for *M *values as a function of the *A *value (*A *= 1/2(log_2 _() +log_2 _())).

## Authors' contributions

AG performed all scanning, image analysis, statistical analysis and wrote the manuscript. HD and TT were involved in discussions on the data analysis and in article preparation. NF developed the program used in this article. LA was involved in discussing the data and assisted with writing the article. MHMG conceived all statistical analysis strategies and associated R programs and wrote the manuscript. All authors approved the final manuscript.

## Supplementary Material

Additional file 1 **Fluctuation, (*e*), of the M values of "outlier" spots within a series of scans for slide #3**. This figure represents the fluctuation (e) of the M values of "outlier" spots within a series of scans for the red channel and the green channel (part A and part B, respectively) and for images obtained with one, two or three acquisitions per line (lines with circles, squares and triangles, respectively). Click here for file

Additional file 2 **The standard deviation of each pixel of "non-outlier" and of "outlier" spots**. This figure shows the standard deviation of each pixel of the spots obtained from 8 successive scans with one, two or three acquisitions per line (from left to right). The height of each vertical bar corresponds to the standard deviation of one pixel within the scan series, graduations are indicated by white broken lines. Part A3, A4 and A5 are for "non outlier" spots and B3, B4 and B5 for "outlier" spots (*F532*_*adj*_). Click here for file
