# Supplementary figures and images for: Characterisation and correction of signal fluctuations in successive acquisitions of microarray images
Source: BMC Bioinformatics. 2009 Mar 30;10:98. doi: 10.1186/1471-2105-10-98 (PMC2681461; doi:10.1186/1471-2105-10-98)

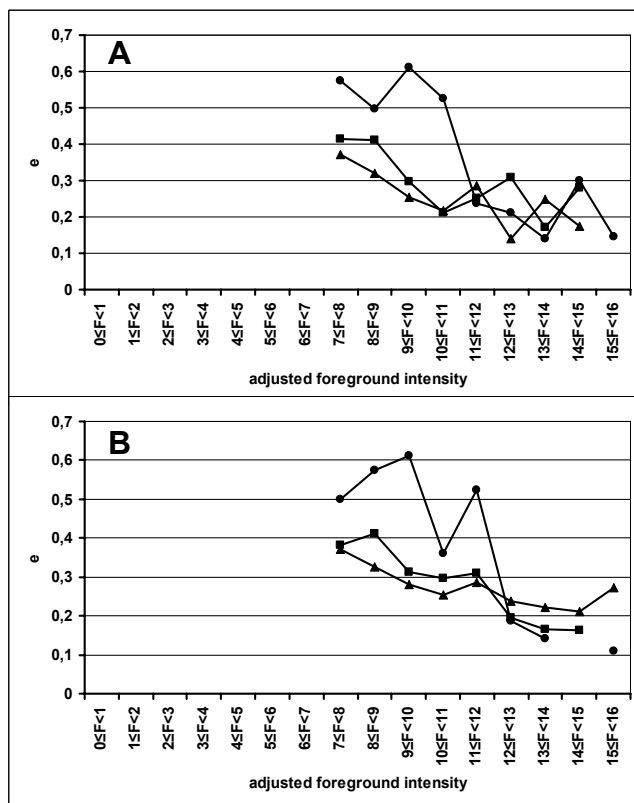

Supplement: Additional file 1 — Fluctuation, (e), of the M values of "outlier" spots within a series of scans for slide #3. This figure represents the fluctuation (e) of the M values of "outlier" spots within a series of scans for the red channel and the green channel (part A and part B, respectively) and for images obtained with one, two or three acquisitions per line (lines with circles, squares and triangles, respectively). [file 1471-2105-10-98-S1.pdf]

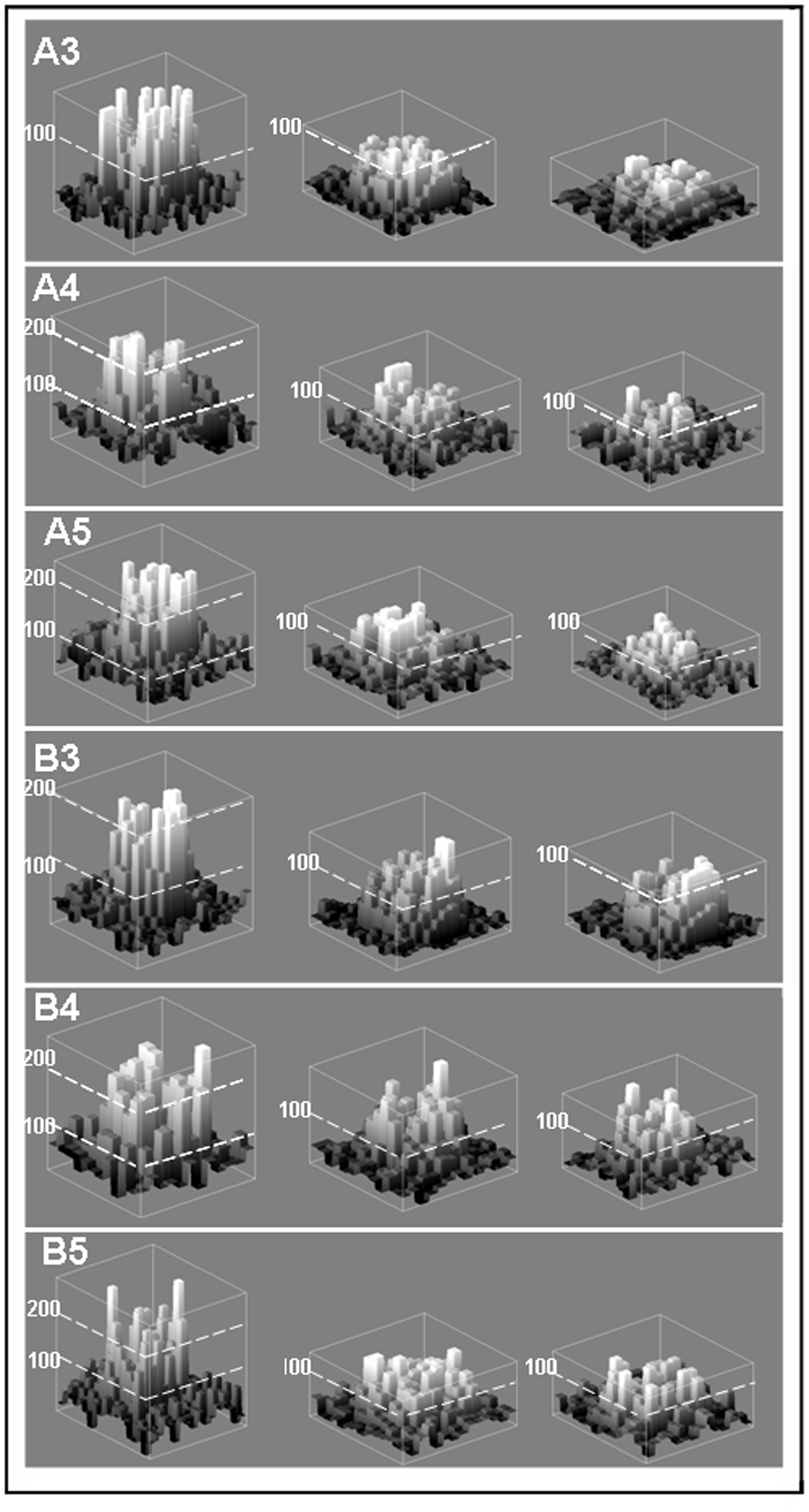

Supplement: Additional file 2 — The standard deviation of each pixel of "non-outlier" and of "outlier" spots. This figure shows the standard deviation of each pixel of the spots obtained from 8 successive scans with one, two or three acquisitions per line (from left to right). The height of each vertical bar corresponds to the standard deviation of one pixel within the scan series, graduations are indicated by white broken lines. Part A3, A4 and A5 are for "non outlier" spots and B3, B4 and B5 for "outlier" spots (F532adj). [file 1471-2105-10-98-S2.tiff]
